# Parents’ and Children’s Emotional Well-Being and Language Beliefs in Heritage Bilingual Families

**DOI:** 10.3390/ejihpe14090166

**Published:** 2024-09-05

**Authors:** Paola Bonifacci, Claudia Borghetti, Martina Cangelosi

**Affiliations:** 1 Department of Psychology, University of Bologna, Viale Berti Pichat 5, 40127 Bologna, Italy; martina.cangelosi2@unibo.it; 2 Department of Modern Languages, Literature and Cultures, University of Bologna, Via Cartoleria 5, 40124 Bologna, Italy; claudia.borghetti@unibo.it

**Keywords:** multilingualism, well-being, parents, children, beliefs

## Abstract

The present study aimed to examine how parents’ psychological characteristics and positive beliefs about multilingualism predict children’s emotional well-being in 51 multilingual families with an immigrant background. Parents were interviewed to assess their beliefs about multilingualism and completed a battery of questionnaires assessing depression, anxiety, psychological distress, parental competence, quality of life, and acculturative stress. They also completed the Strengths and Difficulties Questionnaire (SDQ), which assessed their children’s socio-emotional and behavioral characteristics. The results from regression analyses showed that parents’ depressive symptoms were significant concurrent predictors of children’s conduct problems. In contrast, higher acculturation stress was associated with more emotional problems and better prosociality in children, although the stronger predictor for the latter variable was parents’ self-efficacy. Positive beliefs about bilingualism were not related to children’s well-being. The discussion highlights the importance of targeting parents’ depressive traits and acculturation stress as possible risk factors for children’s emotional and behavioral problems. Conversely, fostering parental self-efficacy may promote children’s prosociality.

## 1. Introduction

Within a bioecological model [1], multiple factors interact to shape children’s emotional well-being, including parent–child relationships, peer contacts, and cultural influences. Well-being is a complex and multidimensional phenomenon that includes physical, psychological, cognitive, social, and economic components [2]. The present paper focuses more specifically on emotional wellbeing, operationalized through a set of psychological factors in parents (i.e., depression, anxiety, psychological distress, parental sense of competence, quality of life, and acculturation stress) and emotional strengths and weaknesses in children [3].

During preschool, children acquire fundamental socio-emotional and behavioral skills that can influence their future social, emotional, and academic achievement and overall well-being (e.g., [4,5]). Social and cognitive development also occurs through interactions with others within specific linguistic and cultural contexts; therefore, linguistic exchanges not only shape the acquisition of language skills, but also convey emotional characteristics and may influence children’s social relationships and emotional well-being [6]. In addition, much evidence, mainly collected in monolingual populations, has reported significant relationships between parent and child well-being in typical populations [7] and in populations with special needs [8,9,10]. To a lesser extent, associations between parent–child well-being have also been studied in multilingual populations [11].

The relationships between parent–child well-being and the role of linguistic beliefs may be different in multilingual families, i.e., families in which at least one parent speaks more than one language, than in monolingual families, on which the literature has mainly focused. If the family’s languages are spoken by a minority group, namely individuals belonging to ethnic, cultural, or linguistic groups that are a minority in the society in which they live, compared to the dominant language of the wider community (Italian in the present study), we also refer to this as heritage bilingualism [12].

The Minority Stress Model refers to the social stress resulting from stigmatized social status and suggests that excessive exposure to social stress may place minority group members at increased risk for adverse mental health outcomes compared to their majority group peers [13]. In fact, heritage bilingual children may be at risk for socio-emotional and behavioral difficulties, especially in preschool years, because their language skills in the school language (i.e., the second language, L2, the one children acquire in addition to their heritage language) may not have reached full proficiency. In addition, there are other factors to consider in comparison to monolingual families, such as attitudes toward bilingualism [14], lower socioeconomic background, and acculturation stress [15], referring to the possible response resulting from adaptation to a host culture (e.g., [16]).

The present study aimed to contribute to better knowledge of the concurrent predictors of child well-being in heritage bilingual families, defined here as families in which at least one parent speaks a language other than the official language of the country of residence and transmits this language to their children. The members of this group are of particular interest to our study because they face unique challenges related to maintaining their heritage language, acculturation stress, and socioeconomic inequalities. It is important to study this population because evidence on the factors underlying emotional well-being in this population is limited and may differ from that of monolinguals or balanced bilinguals who do not have an immigrant background. A better understanding of the family-related factors associated with emotional well-being in heritage bilingual children may also pave the way for implications in educational settings. Another feature of the present study is to consider the role of a positive approach to promoting bilingualism in the home and in the social context. In this regard, a prominent domain to be considered is that of family language policies, which not only refer to language decisions managed within a multilingual family but may also influence children’s well-being.

### 1.1. Family Language Policies and Socio-Emotional Well-Being

Family language policies (FLPs) refer to decisions that multilingual families face about the linguistic upbringing of their children, whether they speak multiple home languages or whether the home languages differ from the institutional language, namely the one which is used within governmental and public institutions, which usually reflects the dominant one. FLPs can be defined based on three main dimensions [17]: beliefs and ideologies (e.g., regarding the social importance of the heritage language), practices (including the adoption of code-switching), and language management (any attempt to direct language use and learning, e.g., the employment of teaching strategies to sustain the children’s heritage language maintenance) (see also [18]) and potentially affect children’s and parents’ well-being [19]. Parental beliefs and ideologies about bilingualism may shape FLPs and thereby influence children’s socio-emotional development.

Indeed, considering beliefs and ideologies, if parents themselves feel societal pressures toward a “monolingual mindset” in the societal context [6] or negative feelings due to the migration process and traumatic experiences, this can lead to the expression of shame, frustration, stress, and anxiety [19]. In one study [20], it was found that a range of non-linguistic factors (demographic, social, and cultural) influence the survival of the heritage language among emigrants. Children might perceive the heritage language spoken at home as an issue of familiar disagreement or that heritage language is devalued or under-recognized as a linguistic skill in the school and societal context. Multilingual children, particularly in the preschool years, might not have acquired full proficiency in L2, particularly in vocabulary and morphosyntactic comprehension, although they may show similar competencies in macro-structural complexity [21]. Due to discrepancies in linguistic competence compared to monolingual children, which may be due to a wide range of sources of variation [22], they might develop frustration, shyness, and difficulties in peer relationships. On the other hand, the insecurity and distress parents feel when their children’s minority language proficiency does not meet their expectations might have a negative impact on children’s well-being [19,23]. For example, parents might force children to respond in the heritage language even though children prefer to use L2.

Contrarily, positive attitudes towards multilingualism and good connections with the heritage language and culture might favor enriched social relationships, emotional well-being, and family cohesion; however, evidence from a narrative review on FLP studies exploring the socio-emotional outcomes shows that these outcomes are underrepresented (see [11]). A review [5] on studies published on the socio-emotional development of multilingual children (aged 0–5 years) found that they have at least equal (if not better) socio-emotional outcomes to native English speakers.

In terms of practice and management, the type of decision a family makes about its language use—for example, whether to use it at home and whether to take steps to pass on a heritage or minority language to children—depends largely on the perceived value of the language from the speaker’s point of view (in terms of beliefs), which in turn can be influenced by broader societal attitudes and ideologies [24]. As stated in [11,25], there may be three main types of conflict related to FLPs: conflicting beliefs of different family members, contradictions between beliefs and practices, and contradictions between practices and expectations. These conflicts among parents could, in turn, affect their daily interactions with children, with negative consequences for emotional attachment, family cohesion, and daily interactions [15].

To conclude, language beliefs, practices, and management might interact with other variables related to parents’ psychological well-being, and more evidence is needed to understand the differential role of FLPs and psychological dimensions on children’s well-being, which will be further defined below.

### 1.2. Parents’ and Children’s Well-Being in Multilingual Populations

In addition to issues related to language choice, parents in multilingual families often have an immigrant background. They may experience more stressors than monolingual and non-migrant families due to financial strain, lack of support, stress of acculturation into a new society, etc. [26].

The previous literature, illustrated in the following sections, has highlighted a number of parental psychological factors that have been found to be related to child well-being, with studies focusing primarily on the following: depressive symptoms, parental self-efficacy, parental distress, and quality of life.

*Depressive symptoms.* The extensive literature on monolingual populations has found that maternal depressive symptoms are associated with poorer emotional well-being in children. A meta-analysis on the relationship between maternal depression and child psychopathology [27] suggests that the effect sizes for the association between mothers’ depression and children’s greater internalizing and externalizing problems were more prominent in ethnic minorities than in the Caucasian sample, together with higher levels of negative affect/behavior. Negative affect was operationalized as the expression of an angry, sad, anxious, or fearful mood. One study [28] found that African American mothers reported higher levels of depressive symptoms than European Americans. However, parental efficacy was significantly associated with depressive symptoms for European American mothers, but not for African American and Hispanic mothers. These studies mainly refer to ethnic minorities, but did not consider multilingualism as a key factor, so they are only informative about cultural and SES differences between the groups.

*Parental self-efficacy.* Parental self-efficacy can be defined as the caregivers’ or parents’ confidence in raising children successfully [29]. Previous studies (for a review see [30]) reported a positive relationship between parental self-efficacy and child behavioral outcomes. Specifically, higher maternal competence was associated with lower levels of aggression and hyperactivity in children [31] and better social skills [32]. However, less evidence has been collected on minority groups. A study [33] found that mothers’ self-efficacy from low-income minority populations was associated with childhood behaviors for healthy childhood growth, including sleep and meal-time media exposure. A systematic review [34] reported mixed results regarding the association between the orientation toward the mainstream culture and parental self-efficacy, with some studies reporting positive [35] or negative [36] relationships. In addition, reduced parental self-efficacy was more common in the early period (first five years) of immigration [37]. Again, these studies do not directly consider the role of multilingualism.

*Parental distress and acculturation stress.* Parental distress refers to the state in which a parent’s perception of the demands of parenting exceeds their resources [38]. In multilingual families, it is often coupled with the demands of parents’ cultural adaptation (acculturative stress) [33], which can be defined as the stress that results from conflict when individuals must adapt to the new culture of the host society [39]. Acculturation stress can arise during the process of adapting to the dominant or mainstream culture and maintaining one’s own culture [40]. Acculturation stressors may include discrimination, economic and employment adversity, language barriers, limited access to health care, changes in family dynamics and culture, and trauma [41]. High levels of acculturation stress are often associated with fewer positive parenting practices [42] and may negatively affect family functioning [43]. One study [44] found that among Mexican mothers, acculturation stress from pregnancy to children’s second birthday negatively predicted children’s socio-emotional and academic outcomes at age 5. In summary, acculturation stress may be a specific parental variable to consider when examining parent–child well-being relationships in multicultural populations.

*Quality of life.* Quality of life can be defined as an individual’s perception of his or her position in life in the context of the culture and value systems in which he or she lives in terms of goals, expectations, standards, and concerns [45]. Despite the multifaceted and complex nature of this construct, some authors suggest measuring it with a single question (e.g., Taking everything in your life into account, please rate your overall quality of life) that respondents choose for themselves by providing a global rating [46,47]. Quality of life relates to well-being, although the former mainly refers to evaluating life circumstances within social norms and values. In contrast, psychological well-being refers more specifically to the subjective evaluation of personal life experiences.

### 1.3. The Present Study

The purpose of the present study was to examine how parents’ psychological attributes and positive beliefs about multilingualism predict child well-being in a sample of heritage bilingual families.

Specifically, we included parent questionnaires on depressive and anxiety traits, psychological distress, parental sense of competence, quality of life, and an acculturation stress inventory. In addition, beliefs about bilingualism in the family were coded based on interviews about family language policies. Children’s well-being and behavioral characteristics were assessed using the Strengths and Difficulties Questionnaire (SDQ) [3], which assesses emotional problems, conduct problems, impulsivity/hyperactivity, peer relationship problems, and prosociality.

The main aims of the study were the following:

(1) As a first aim, the study examines the relationships between parental psychological dimensions and beliefs about bilingualism. Based on the previous literature, we expect positive beliefs about multiculturalism to be associated with lower acculturation stress and negative affect, but with higher parental self-efficacy (e.g., [6,11]).
(2) Second, the study aims to examine concurrent relationships between parental psychological characteristics and beliefs about multilingualism and child well-being. We expect that parental depression traits (and negative affect) and acculturation stress will be associated with increased emotional, behavioral, and peer problems (e.g., [26,27,28]) and that parental self-efficacy will be associated with better child well-being (e.g., [29,30,31]).

## 2. Materials and Methods

### 2.1. Participants

The study was conducted in Italy and included 51 multilingual families (Mothers M age = 39.63; SD = 5.29: Fathers M age = 43.86; SD = 6.23; average education level for each couple M = 4.66 according to the Hollingshead criteria with scores ranging from 1 = elementary school to 7 = PhD or Post Lauream Specialization). At least one parent in each couple reported speaking a native language other than Italian and/or came from a country other than Italy (71% of fathers; 94% of mothers). Children (M age = 4.74, SD = 0.56) have been recruited from public infancy schools in Bologna, a city located in northern Italy. Correlations of parents’ age and education level with parents and children’s measures did not reveal significant patterns (all *p* > 0.01); therefore, we did not include these variables as covariates in regression analyses (see below).

As a first step, sample selection has been based on a parental questionnaire, consisting of an Italian adaptation of the Language and Social Background Questionnaire (LSBQ) [48], where both parents had to report on their sociolinguistic background and their daily usage habits. Based on the inclusion criteria and the willingness of the families to participate, 51 heterosexual couples were involved in a telematic interview that lasted about half an hour and explored parental beliefs and ideologies regarding multilingualism. Both parents were not always contemporarily present for the whole interview due to work or time constraints, but information about both was collected through the parents involved. Also, 51 fathers and mothers completed the emotional questionnaires. In case of missing values (less than 5%), we considered the score of the single parent. More details on the language distribution can be found in Table 1.

### 2.2. Materials

The parent questionnaires were administered using the survey platform Qualtrics. If both parents completed the survey, their responses were averaged. Some questionnaires (see below) were translated for the present study and adapted to be culturally relevant and consistent with the Italian context. The translation did not follow a strict protocol of back translation, but the authors cross-checked the readability and clarity of each item.

*Depression Anxiety Stress Scale—DASS* [49]. This questionnaire is a non-clinical measure to assess symptoms of depression, anxiety, and stress during the previous week. It consists of three subscales, one for each of the three dimensions mentioned above. It has a total of 21 items, 7 for each subscale. Participants can choose their responses from 4 options on a Likert scale ranging from 0 = does not apply to me at all to 3 = applies to me a great deal or most of the time. The Italian adaptation has demonstrated reliable psychometric properties [50]. The DASS has also been found to be a reliable instrument in various cultural contexts [51]. Scores range from 0 to 21. The Cronbach alpha reliability for the depression scale is 0.91, for the anxiety scale it is 0.84, and for the stress scale it is 0.90.

*Riverside Acculturative Stress Index—RASI* [52]. This questionnaire aims to assess acculturative stress in individuals undergoing a process of adaptation to a new cultural environment. It consists of a series of 15 items that inquire about various aspects of acculturative stress, such as language barriers, discrimination, cultural conflicts, and feelings of homesickness, in five areas: work challenges, language skills, intercultural relations, discrimination, and cultural isolation. The questionnaire was translated for the present study. Scores ranged from 15 to 75. The Cronbach alpha reliability for the entire scale is 0.93.

*Parenting Sense of Competence Scale—PSOC* [53]. This questionnaire is designed to assess parents’ perceptions of their competence as caregivers for their children. It consists of 17 item that explore different facets of parenting, such as feelings of satisfaction, efficacy, and perceived difficulty as a caregiver. Parents can respond by choosing from 6 options on a Likert scale ranging from 1 = strongly disagree to 6 = strongly agree. The questionnaire was translated for the present study. Scores ranged from 17 to 102. The Cronbach alpha reliability for the entire scale is 0.81.

*Strengths and Difficulties Questionnaire—SDQ* [3]. The single-sided version of the Italian parent version of the SDQ was administered to both mothers and fathers, who were asked to rate their children’s strengths and weaknesses. The questionnaire contains 25 items describing positive and negative behavioral traits in children, and it provides five subscales: emotional symptoms, conduct problems, hyperactivity–intention, peer problems, and prosocial behavior, each with five items and a score ranging from 0 to 10. Respondents use a three-point Likert scale (0 = not true, 1 = somewhat true, and 2 = definitely true). Higher scores on the first four subscales indicate difficulty, and a higher total score reflects more severe problems, whereas higher scores on the fifth indicate better social behavior. The Italian version of the SDQ has shown an acceptable internal consistency for all the scales [54]. The SDQ has also been found to be a reliable instrument in diverse cultural contexts [55]. The Cronbach alpha reliability for the emotional symptoms scale is 0.74; for the conduct problems scale, it is 0.70; for the hyperactivity scale, it is 0.77; for the peer problems scale, it is 65, while for the prosocial behavior scale, it is 0.78.

*World Health Organization Quality of Life—WHOQOL* [45,47]. For the present study, we included a single question from the questionnaire: “How would you rate your quality of life?”. Scores ranged from 1 (very poor) to 5 (very good). The Cronbach’s alpha reliability for the entire scale is 0.89.

*Parental interviews.* The interview transcripts were analyzed thematically [56] using NVivo [57]. Initially, the so-called ‘Grounded Theory Method’ was used (e.g., [58]. Thus, apart from the inevitable influence of the aims of the study, it was the systematic analysis of the participants’ words—rather than the need to test a given hypothesis—that led to the definition of specific conceptual categories. Parents’ statements were coded through several rounds of analysis by one of the authors and an additional annotator, regardless of the specific question the participants were answering. Then, doubtful cases were discussed and solved among the authors. We reported a “0” if a particular topic was not present in the family’s discourses and a “1” if it was addressed at least once. This bottom-up approach paved the way for a more focused analysis, as respondents’ positive beliefs about bilingualism were aggregated to identify general trends within and across families. Examples of the items that make up the index of positive beliefs about bilingualism are as follows: perceived societal valorization of bilingualism, parents’ positive beliefs about the mother tongue, beliefs about the benefits of growing up bilingual, and parents’ positive beliefs about speaking two or more languages. Scores greater than 1 were possible if parents reported the same issue in more than one thematic area. Scores ranged from 1 to 12. The index of positive beliefs had a mean score of 6.07 occurrence (*SD* = 2.78).

### 2.3. Procedure

The parent interviews were conducted using the Microsoft Teams online platform. The sessions lasted about 30 min for each family. In most cases, both parents participated. Each interview was conducted by an experimenter following a semi-structured interview protocol. The interviews were recorded and later transcribed. After each interview, the questionnaires were administered to the parents.

The present research project was conducted in accordance with the ethical standards of the University of Bologna Ethics Committee. The project has been assigned Protocol Number 0250367, Date: 17 October 2022.

### 2.4. Data Planning

All the analyses reported are run using RStudio [59] and MuMin [60] as external packages.

In Table 2, we report descriptive statistics for parents and children for all questionnaires included in the study. Both parents’ scores were averaged to obtain an overall view of the family, reducing individual bias and better reflecting the family environment.

Pearson correlation analyses were performed to assess relationships between parental variables and to consider possible multicollinearity in our models; furthermore, we performed Variance Inflation Factor analyses considering the threshold of 5 to include predictors in our models.

Finally, five linear regression analyses were conducted to examine how parental emotional and psychological well-being and their perceptions of bilingualism are related to bilingual children’s socio-emotional characteristics, reflected by children’s scores on the SDQ scales.

Our final models include the SDQ subscales (i.e., conduct, emotion, hyperactivity, peer relations, and prosocial behavior) as dependent variables. Each model included the following predictors referred to in the parental measures: DASS scales (i.e., depression, anxiety, and stress), Riverside Acculturation Stress Index, WHOQOL, and parental sense of competence scale results and positive beliefs about bilingualism. Raw scores were considered (see score range in the Materials and Methods section).

## 3. Results

### 3.1. Research Question 1—Relationships between Parental Psychological Dimensions and Beliefs about Bilingualism

Correlation patterns between parental questionnaires and beliefs are reported in Table 3. In the correlation analyses, we set the level of significance at *p* < 0.01.

Correlation analyses evidenced, as main patterns, a negative relationship between acculturation stress (RASI) and parental self-efficacy (PSOC) and a positive relationship between acculturation stress and anxiety (Dass anxiety).

### 3.2. Research Question 2—Concurrent Relationships between Parental Psychological Dimensions and Beliefs about Multilingualism and Children’s Well-Being

Considering the SDQ emotion scale, the regression model [*F* (7,45) = 3.17, *R^2^* = 0.226] evidenced that parental scores on the Riverside Acculturation Stress Index (RASI) were significant predictors (*p* < 0.01), with a positive estimate (estimate = 0.058). This means that higher parental acculturation stress scores are significantly associated with higher rates of emotional problems in children. Please note that an estimate value of 0.058 means that a 1 unit increase in the RASI score is associated with a 0.058 increase in children’s emotional characteristics. The other variables of interest do not significantly explain children’s conduct (See Table 4 for more detailed results on this dimension).

Regarding the SDQ conduct scale, the regression model [*F* (7,45) = 3.40; *R*^2^ = 0.244] showed that parental scores on the DASS depression scale played a significant role (*p* < 0.05), with a positive estimate (estimate = 0.140), meaning that higher parental depression scores are significantly associated with higher rates of child conduct problems. The other variables of interest do not significantly explain children’s conduct problems (see Table 5 for more detailed results on this dimension).

Considering the regression model on the SDQ hyperactivity scale [*F* (7,44) = 1.76, *R*^2^ = 0.094], it was found that none of the variables of interest significantly explain children’s hyperactivity attributes (See Table 6 for more detailed results on this dimension).

Considering the SDQ peer relation scale, the regression model [*F* (7,45) = 2.57, *R*^2^ = 0.174] showed that RASI parental results exerted a significant role (*p* < 0.001), with a positive estimate (estimate = 0.049), meaning that higher parental scores in terms of their general happiness and satisfaction about their lives are marginally protective towards their children’s peer relations problems.

The other variables of interest do not significantly explain children’s conduct scores (See Table 7 for more detailed results on this dimension).

Considering the SDQ prosocial behavior scale, the model [*F* (7,44) = 3.08, *R*^2^ = 0.222] evidenced that RASI parental results exerted a significant role (*p <* 0.05), with a positive estimate (estimate = 0.041), meaning that higher parental scores in terms of their acculturation stress are associated with children’s higher scores in terms of prosocial behavior.

Furthermore, the PSOC is significantly predictive of higher rates in terms of prosocial behavior (*p* < 0.05), with a positive estimate (estimate = 0.070), meaning that a higher parental sense of competence is predictive of higher prosocial behavior skills for their children.

The other variables of interest do not significantly explain children’s conduct attributes (See Table 8 for more detailed results on this dimension).

## 4. Discussion

The present study was conducted on a sample of multilingual families living in Italy and who speak a language other than Italian in their home context. The aims of the study were twofold. First, as an exploratory goal, the relationships between different dimensions of parents’ psychological dimensions and beliefs about bilingualism were examined. Second, regression models were developed to analyze how parental psychological dimensions and beliefs about bilingualism concurrently predict children’s well-being as measured by the SDQ [3]. The original contribution of the study is to examine multiple concurrent predictors of child well-being in multilingual families, a vulnerable group at higher risk for psychological distress. More specifically, to our knowledge, this is the first study to link parents’ psychological factors (i.e., depression, anxiety, psychological distress, parental competence, quality of life, and acculturation stress) and beliefs about multilingualism with children’s emotional and behavioral skills.

Correlational analyses suggest a negative relationship between acculturation stress and parental self-efficacy, and a positive relationship between acculturation stress and anxiety. These findings suggest that even without clear causal relationships, acculturation stress is associated with poor parental self-efficacy, consistent with [42], who reported that acculturation stress was associated with reduced positive parenting practices. Regarding the association between anxiety symptoms and acculturation stress, there might be a bidirectional relationship, given that parents with anxiety traits might be less able to implement efficient coping strategies to reduce the burden of acculturation processes. On the other hand, facing challenges related to the acculturation process might increase anxiety traits.

Surprisingly, positive beliefs about bilingualism were not significantly related to parents’ psychological dimension. However, from a qualitative perspective, given the relatively limited sample size of the study, there was a trend toward a negative correlation with depression (r = −0.226, *p* = 0.056) and a positive relationship with parental self-efficacy (r = 0.244, *p* < 0.05). While the association with depression could be read as a socio-emotional effect of coping with bilingualism [11], including beliefs about it, the association with self-efficacy would require further investigation. If confirmed, this could suggest the importance of supporting parents’ positive views of multilingualism and self-efficacy to promote a positive interaction between these two dimensions. In other words, the more parents feel competent in their role, the more they may overcome their fears about raising multilingual children, and vice versa.

Turning to the analysis of how parents’ psychological characteristics and beliefs about multilingualism concurrently predict children’s well-being, a multifaceted picture emerged.

First, it was found that depression traits in parents predicted conduct problems in children. A previous study [61] in monolingual populations reported that parental depressive traits could have consequences for interactions with children, such as more physical punishment, verbal aversion, and less warmth monitoring. In turn, coercive interactions may lead children to engage in more behavioral problems. In the present study, we did not directly address parental behavior; therefore, we cannot clearly support this pattern. However, further studies could better investigate these associations.

Second, parental self-efficacy positively predicted children’s prosociality. Previous research has already demonstrated the effects of parental self-efficacy on better social competence in the monolingual population [32]. The present study also adds positive evidence regarding heritage bilingual families.

The third main pattern showed that higher acculturation stress predicted increased emotional and peer relationship problems in children. Given that acculturation stress has been reported to be associated with fewer positive parenting practices (i.e., parents’ expressions of warmth, reasoning, and control) [42] and a negative impact on family functioning [43], this may suggest that a child’s emotional development and social behaviors with their peers can be negatively affected too. However, to our knowledge, only one previous study has directly assessed the relationship between parental acculturation stress and children’s emotional well-being in multilingual families [44].

A peculiar finding of the present study was that, despite increased peer relationship difficulties in children from families with higher acculturation stress, they also exhibited higher prosocial behaviors. This finding may seem counterintuitive or contradictory. However, we can speculate on how we think that this finding might be coherent in the larger picture. Prosociality refers to behaviors that are intended to benefit others, including comforting, sharing, and helping attitudes. Therefore, engaging in prosocial behaviors does not necessarily mean having good peer relationships. Prosocial behavior can also be seen as a personal disposition to be responsive to the needs of others and as a way of connecting with others. Therefore, children who experience difficulties and stresses in the family environment may develop better abilities to empathize with others. In another study in a larger community sample (authors, submitted), we found that multilingual children had more peer relationship problems and higher prosociality compared to their monolingual peers. In summary, the co-occurrence of peer relationship problems and increased prosociality is an innovative contribution of the present study that needs to be further explored in future research.

## 5. Conclusions

In summary, the present study revealed some patterns consistent with previous studies of monolingual populations and strengthened the possibility to extend the findings to multilingual populations. It was shown that parents’ depression traits were concurrent predictors of children’s behavior, and that parents’ self-efficacy was related to children’s prosocial behavior. In particular, acculturation stress was associated with children’s emotional and peer problems, but also with increased prosociality. On the other hand, we found no clear evidence of an impact of positive beliefs about parents’ bilingualism on children’s socio-emotional attributes.

Some limitations that may limit the generalizability of the results need to be acknowledged. First, the sample is relatively small, and the study is cross-sectional; longitudinal studies with larger samples would be needed to understand predictive patterns. The second point is that for the present study, we coded the interviews based on a bottom-up approach, which led to the identification of mainly positive beliefs about multilingualism. A different coding approach based on a top-down extraction of more thematic units that isolate a broader picture of the phenomena may be a promising approach for future research. It would also be interesting to include dimensions related to children’s agency in language choice and how this relates to their socio-emotional skills. Finally, also due to the relatively limited sample, we did not analyze the differential role of each parent’s psychological characteristics, and some measures did not have Italian validation. However, unlike previous studies that mainly included mothers, the present studies included, for most measures, the mean scores derived from both parents’ questionnaires.

In conclusion, the present study provides an original picture of parent–child socio-emotional characteristics in multilingual populations, and we could speculate about possible implications for educational settings. For example, it is important to support parents of multilingual children with conduct problems, emotional problems, and difficulties in peer relationships, because in many cases they may have depressive traits or low self-efficacy. This could be achieved through parenting programs for immigrant parents, which have been shown to have a positive impact on positive parenting behaviors, parent–child relationships, and parenting stress [62].

## Figures and Tables

**Table 1:** Languages spoken other than Italian.

Languages	Mothers	Fathers
Albanian	3	3
Arabian	4	3
Bengali	5	4
Chinese	1	0
Creole	1	1
Filipino	2	2
French	2	0
Greek	1	1
English	1	1
Moldovan	2	2
Portuguese	1	0
Romanian	8	7
Russian	3	0
Slovak	1	0
Spanish	2	2
Tigrinya	1	1
Ukrainian	0	1
Urdu	1	1

**Table 2:** Descriptive statistics for parents and children in the main measures included in the sample.

Measures			Mean	SD
Parents	Dass	Depression	17.92	4.78
		Anxiety	16.78	3.75
		Stress	22.24	5.72
	RASI		30.65	12.10
	WHOQOL		4.00	0.60
	PSOC		67.75	9.02
Children	SDQ	Conduct	2.00	1.84
		Emotion	1.63	1.47
		Hyperactivity	3.27	1.82
		Peer relations	4.92	1.11
		Prosocial behavior	7.88	1.62

**Table 3:** Correlations among parents’ questionnaires and beliefs about bilingualism.

	Positive Beliefs Bilingualism	Dass Depression	Dass Anxiety	Dass Stress	PSOC	WHOQOL
Dass Depression	−0.22 #					
Dass Anxiety	0.00	0.68 **				
Dass Stress	0.05	0.51 **	0.66 **			
PSOC	0.24 *	−0.11	−0.21	−0.25		
WHOQOL	0.05	−0.15	−0.14	0.02	0.27	
RASI	−0.01	0.25	0.40 **	0.19	−0.39 **	−0.24

** *p* < 0.01; * *p* < 0.05; # *p* = 0.056.

**Table 4:** Role of parental emotional and psychological well-being and their positive perception about being bilinguals on children’s emotion attributes.

	Estimate	Std. Error	*t* Value	*p* Value
Intercept	−2.1357	1.97741	−1.080	0.28586
Dass Depression	0.00088	0.05588	0.016	0.98751
Dass Anxiety	0.02086	0.08278	0.252	0.80219
Dass Stress	0.03181	0.04502	0.707	0.48345
RASI	0.05758	0.01731	3.325	0.00176 **
WHOQOL	0.51983	0.32608	1.594	0.11790
PSOC	−0.01908	0.01808	−1.055	0.29699
Pos. B. Biling.	0.01239	0.07301	0.170	0.86605

** *p* < 0.01.

**Table 5:** Role of parental emotional and psychological well-being and their positive perception about being bilinguals on children’s conduct attributes.

	Estimate	Std. Error	*t* Value	*p* Value
Intercept	0.739654	2.430769	0.304	0.7623
Dass Depression	0.139820	0.068697	2.035	0.0477 *
Dass Anxiety	−0.018924	0.101754	−0.186	0.8533
Dass Stress	0.042903	0.055341	0.775	0.4422
RASI	0.030780	0.021284	1.446	0.1551
WHOQOL	−0.534304	0.400846	−1.333	0.1893
PSOC	−0.008154	0.022230	−0.367	0.7155
Pos. B. Biling.	−0.026051	0.089746	−0.290	0.7729

* *p* < 0.05.

**Table 6:** Role of parental emotional and psychological well-being and their positive perception about being bilinguals on children’s hyperactivity attributes.

	Estimate	Std. Error	*t* Value	*p* Value
Intercept	2.991428	2.756139	1.085	0.284
Dass Depression	0.059547	0.077452	0.769	0.446
Dass Anxiety	−0.004614	0.113073	−0.041	0.968
Dass Stress	0.070611	0.062584	1.128	0.265
RASI	0.021099	0.023650	0.892	0.377
WHOQOL	−0.670364	0.448280	−1.495	0.142
PSOC	0.002191	0.025449	0.086	0.932
Pos. B. Biling.	−0.070732	0.100492	−0.704	0.485

**Table 7:** Role of parental emotional and psychological well-being and their positive perception about being bilinguals on children’s peer relation attributes.

	Estimate	Std. Error	*t* Value	*p* Value
Intercept	−1.194425	1.783262	−0.670	0.50641
Dass Depression	−0.006566	0.050397	−0.130	0.89692
Dass Anxiety	0.048644	0.074649	0.652	0.51795
Dass Stress	−0.008245	0.040599	−0.203	0.83998
RASI	0.048917	0.015615	3.133	0.00304 **
WHOQOL	0.457445	0.294069	1.556	0.12682
PSOC	0.032400	0.016308	1.987	0.06306
Pos. B. Biling.	−0.000621	0.065840	−0.009	0.99252

** *p* < 0.01.

**Table 8:** Role of parental emotional and psychological well-being and their positive perception about being bilinguals on children’s prosocial behavior attributes.

	Estimate	Std. Error	*t* Value	*p* Value
Intercept	−1.05920	2.44327	−0.434	0.6668
Dass Depression	0.07680	0.06245	1.230	0.2253
Dass Anxiety	−0.03111	0.09223	−0.337	0.7374
Dass Stress	−0.04919	0.05004	−0.983	0.3309
RASI	0.04166	0.02000	2.083	0.0431 *
WHOQOL	0.58700	0.36541	1.606	0.1153
PSOC	0.06961	0.02624	2.653	0.0111 *
Pos. B. Biling.	0.13710	0.08373	1.637	0.1087

* *p* < 0.05.

## Data Availability

The data presented in this study are available on request from the corresponding author. The data are not publicly available due to privacy issues.
